# Progress in Neurology

**Published:** 1903-05-23

**Authors:** 


### PROGRESS IN NEUROLOGY.

**Histology of the Motor Cortex.**—Farrar[1] has recently given an able review of the various investigations which have defined the structure and limits of the motor cortex. It is characterised by its unusual width, dependent on the marked development of the medium-sized pyramidal cell layer containing the specific plexus of Cajal, by the absence of a clearly defined granular zone, and by the presence of giant cells in the sixth layer with thicker and denser radial bundles and a richer development of the overlying tangential fibres which are of maximum density in those portions of the convolution which contain the greatest number of giant cells. Passing beyond the limits of the motor area one observes a gradual change in the cortical type, the third layer becoming narrower, the fifth narrower, and the specific elements of the sixth disappearing. This change is typically illustrated in a cross section through the anterior and posterior central convolutions. These histological results accurately agree with those obtained by experimental stimulation, for whilst earlier experimenters thought that the motor area embraced nearly a half of the brain surface, the most recent—Sherrington and Grünbaum—limit it to the precentral convolution.

**Sensation in Motor Paralysis of Cerebral Origin.**—Whilst some observers hold that there are separate cortical areas for motor and sensory functions, others think that the so-called "motor cortex" is in reality a sensori-motor one. A contribution to this question has been made by Gordon,[2] who examined the condition of sensation in 35 cases of hemiplegia of cerebral origin, in whom mental functions were intact. It was found that in the largest number of cases the upper extremity is affected with sensory disturbances—this is true, for each of the three forms of sensation—for pain, temperature, and touch. In the lower limb, touch and pain are affected in a slightly smaller number of cases than on the trunk, except the temperature sense, which suffers more in the leg than in the trunk. The face is the least affected. Anæsthesia is met with in a comparatively small number, hypo-sensation takes the largest number of cases, whilst hyperæsthesia is found in extremely few. The disturbed sensations have a tendency to become normal from the distal end of the limb towards the root. The stereognostic sense was disturbed in 29 out of the 35 cases, and in 22 the loss was complete. The greater the involvement of the other sensations the more marked was astereognosis, a fact which favours the view that the stereognostic sense is dependent on the integrity of the three cardinal sensations. The sense of posture for the upper limb was involved in but 17 cases out of the 35, a fact which leads to the idea that disturbances of other sensations do not necessarily involve the posture sense. There is a certain parallelism between the degree of motor and of sensory impairment, in those cases in which the motor paralysis is not marked the sensory impairment is also less marked. The sensory impairment tends to become less in the course of years. The conclusion drawn is, that hemisensory disturbances probably always accompany a motor paralysis of cerebral origin.

**Paradoxical Pseudo-hypertrophy following Infantile Cerebral Hemiplegia** is a very rare condition, of which some new cases have been reported by Pierce Clark.[3] Infantile cerebral hemiplegia is usually followed by a more or less marked degree of muscular and bone atrophy, but exceptionally the limbs may become larger. The initial symptoms of such cases have been the usual ones of convulsions, vomiting, fever and physical prostration, followed by hemiplegia more or less marked. The face and leg have recovered, whilst the arm was left partially paralysed. Athetosis is almost universally present, though in a few cases a severe partial epilepsy was present instead. The enlargement has had its seat in the upper extremity in every case but one reported, whilst in that the leg was affected. The muscles most frequently hypertrophied are found in the following order of occurrence: biceps, deltoid and triceps. The bones of the entire upper extremity may be hypertrophic, although the bones usually found enlarged are those corresponding to the hypertrophic soft parts they underlie. The hypertrophic condition is probably due to the post-hemiplegic disorder of motility.

[1] Amer. Jour. of Insanity, Jan. 1903. [2] Jour. of Nerv. and Ment. Dis., March, 1903. [3] Ibid., Nov. 1902.

*(To be continued.)*

